# Overcoming Resistance to Immune Checkpoint Blockade in Liver Cancer with Combination Therapy: Stronger Together?

**DOI:** 10.1055/a-2334-8311

**Published:** 2024-06-21

**Authors:** Wiebke Werner, Maria Kuzminskaya, Isabella Lurje, Frank Tacke, Linda Hammerich

**Affiliations:** 1 Department of Hepatology and Gastroenterology, Charité Universitaetsmedizin Berlin, Berlin, Germany

**Keywords:** hepatocellular carcinoma, cholangiocarcinoma, immunotherapy, checkpoint inhibitors, resistance

## Abstract

Primary liver cancer, represented mainly by hepatocellular carcinoma (HCC) and intrahepatic cholangiocarcinoma (CCA), is one of the most common and deadliest tumors worldwide. While surgical resection or liver transplantation are the best option in early disease stages, these tumors often present in advanced stages and systemic treatment is required to improve survival time. The emergence of immune checkpoint inhibitor (ICI) therapy has had a positive impact especially on the treatment of advanced cancers, thereby establishing immunotherapy as part of first-line treatment in HCC and CCA. Nevertheless, low response rates reflect on the usually cold or immunosuppressed tumor microenvironment of primary liver cancer. In this review, we aim to summarize mechanisms of resistance leading to tumor immune escape with a special focus on the composition of tumor microenvironment in both HCC and CCA, also reflecting on recent important developments in ICI combination therapy. Furthermore, we discuss how combination of ICIs with established primary liver cancer treatments (e.g. multikinase inhibitors and chemotherapy) as well as more complex combinations with state-of-the-art therapeutic concepts may reshape the tumor microenvironment, leading to higher response rates and long-lasting antitumor immunity for primary liver cancer patients.

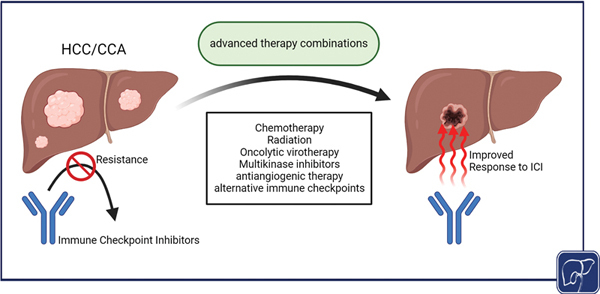

## Immune Checkpoint Inhibitors in Primary Liver Cancer

### The Concept of Disturbed Immune Surveillance in the Cancer Immune Cycle

**Fig. 1:**
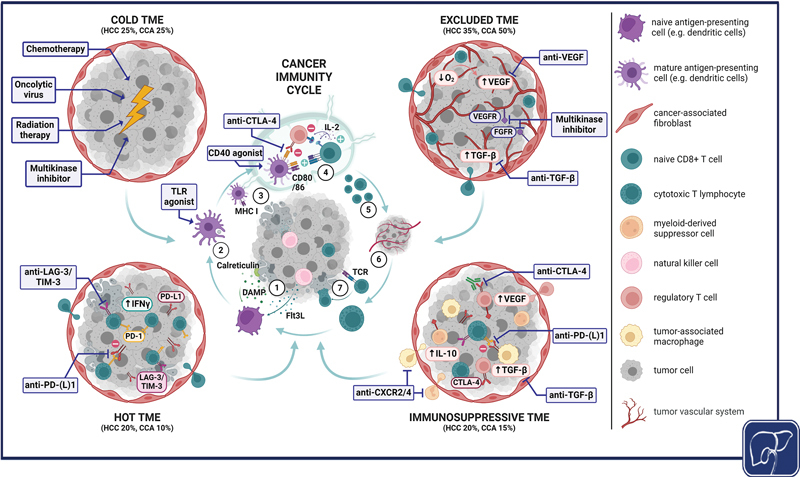
Reshaping the tumor microenvironment (TME) to reestablish immunosurveillance in primary liver cancer. During the cancer immunity cycle, 26 immunogenic cell death (ICD) and cells of innate immunity recruit professional antigen-presenting cells (APC) to the tumor (1). APC process and present tumor-associated antigens (TAA) during their maturation (2) and relocate to the tumor-draining lymph node (3), where they cross-present and prime naive cytotoxic T lymphocytes (CTL) (4). Following clonal expansion (5), TAA-experienced activated CTL migrate to the tumor and infiltrate the TME (6), where they recognize and kill tumor cells (7). Various mechanisms of tumor immune escape are implemented in the TME, which is represented in four different schematic manifestations (based on Galon and Bruni 50 ). While the *hot* TME (lower left) shows high CTL infiltration, programmed death ligand 1 (PD-L1) expression and IFN-γ signaling, *cold* TME (upper left), display near to no CTL infiltration or PD-L1 expression. The *excluded* TME (upper right) is rich in cancer-associated fibroblasts and T cells in the periphery but not in the tumor center, and the *immunosuppressive* TME (lower right) shows heightened infiltration of immunosuppressive cells. TME frequencies in HCC and CCA are based on Job et al and Giraud et al. 214 243 Established and experimental cancer therapies combined with immune checkpoint inhibitor therapy (blue boxes) may alter the TME and facilitate reentry into the cancer-immunity cycle. (Created with biorender.com.) CAF, cancer-associated fibroblasts; CCA, cholangiocarcinoma; CTLA-4, cytotoxic T-lymphocyte-associated protein 4; CXCR, C-X-C-chemokine receptor; DAMP, danger-associated molecular patterns; Flt3L, FMS-like tyrosine kinase 3 ligand; IL, interleukin; FGFR, fibroblast growth factor receptor; HCC, hepatocellular carcinoma; LAG-3, lymphocyte-activation gene 3; MHC, major histocompatibility complex; MKI, multikinase inhibitor; PD-(L)1, programmed death (ligand) 1; TGF-β, tumor growth factor-beta; TIM-3, T cell immunoglobulin and mucin-domain-containing molecule 3; TLR, toll-like receptor; VEGF(R), vascular endothelial growth factor (receptor).

Paul Ehrlich suggested a potential tumor-controlling role of the immune system, which has been formally introduced as *cancer immune surveillance* in the 1950s. 22 At that time, this hypothesis was abandoned by the scientific community due to lack of evidence but revived in a hallmark review from 2002, which evolved this theory and described the development of neoplasms despite a functioning host immune system as *cancer immunoediting* . 23 During elimination phase or immune surveillance, the immune system is able to eradicate degenerated cells by careful orchestration of innate and adaptive immune responses, mainly mediated by CD8+ T lymphocytes or cytotoxic T cells (CTLs; Fig. 1 ). 23 Briefly, the (immunogenic) death of tumor cells releases tumor-associated antigens (TAA) and danger-associated molecular patterns (DAMP). Following antigen uptake and processing, activated antigen presenting cells (APC) migrate to the draining lymph nodes (or to tertiary lymphoid structures), where they cross-present the antigens and prime CTL. Activated CTL then travel back to the tumor and kill tumor cells by antigen-mediated cytotoxicity. Other lymphocyte subsets, such as natural killer (NK) cells 24 and CD4+ T cells, 25 can exert or support antitumor cytotoxicity as well. Each of these individual steps can be influenced and modified by precancerous alterations, making them *rate-limiting* for a successful antitumor response. 26 Due to selection pressure, less immunogenic cancer cells evolve and escape the immune system, further expanding with the help of additional immunosuppressive adaptations. 23

### Commonly Applied Immune Checkpoint Inhibitors in Cancer Immunotherapy

Inhibitory immune checkpoints are important regulators of the immune system, hindering excessive immune responses by putting a break on T cell–mediated adaptive immune functions and facilitating self-tolerance. 27 However, T cell–mediated cytotoxicity against cancer cells can also be prevented by the same mechanisms. 28

The best studied immune checkpoints to date are CTLA-4 and the PD-1/PD ligand 1 (PD-L1) axis. 29 30 Activated T cells express CTLA-4 on their surface, where it competes with CD28 for B7 ligands CD80/86 on APC. 30 31 While binding of CD28 has a costimulatory effect, ensuring activation and differentiation of T cells, CTLA-4 functions as an inhibitor with far higher affinity for CD80/CD86 than CD28. 32 33 CTLA-4 has two modes of action in the cancer immunity cycle, which ultimately lead to immunosuppression: during T cell priming in the draining lymph node, antigen recognition of naive T cells is hindered by undermining the costimulatory signal mediated by CD80/CD86 and regulatory T cells (T _reg_ ), which constitutively express CTLA-4, inhibit costimulatory signals on dendritic cells (DC) leading to anergy during antigen presentation and reduced T cell priming ( Fig. 1 ). Additionally, CTLA-4-expressing T _reg_ hampers antigen-mediated T cell killing in the TME ( Fig. 1 ). Blocking CTLA-4 with monoclonal antibodies revives the costimulatory signal necessary for T cell activation and induces elimination of T _reg_ by antibody-dependent cellular cytotoxicity. 30 34 Ipilimumab was the first FDA-approved CTLA 4-inhibitor for solid cancers (i.e., melanoma). 14

The PD-1 receptor is expressed on activated T and B cells, NK cells, and monocytes, and produces a negative signal cascade when binding to its ligands PD-L1/L2 on APC and tumor cells, inhibiting T and B cell receptor signaling, cytokine production, and production of proapoptotic proteins. 30 35 Especially chronic antigen stimulation by uninterrupted carcinogenesis can cause upregulation of PD-1 and other checkpoints, consequently leading to loss of T cell effector functions (called T cell exhaustion) and further assisting cancer immune escape ( Fig. 1 ). 36 Treatment with antibodies against PD-1 (e.g., nivolumab, pembrolizumab) or PD-L1 (e.g., atezolizumab, durvalumab) prevents binding of the natural ligands, inhibiting the immunosuppressive function of PD-1 and ensuring proper T cell function. 30 37

Additionally, alternative checkpoints like lymphocyte activation gene-3 (LAG-3) and T cell immunoglobulin and mucin domain-containing molecule 3 (TIM-3) have become increasingly interesting in the treatment of solid tumors. 38 LAG-3 and TIM-3 are transmembrane proteins expressed by activated T cells and have a similar function to PD-1 and CTLA-4. While LAG-3 exerts the inhibitory function either as a ligand for MHC class II or fibrinogen-like protein 1, 39 TIM-3 is also expressed by other immune cell types such as DC, T _reg_ , and NK cells, and activates inhibitory signaling through interactions with HLA-B-associated transcript 3 and galectin 9. 40 Clinical trials investigating the combination of both checkpoints are ongoing. 38 41

### Monotherapy Trials for Primary Liver Cancer

The first ICI monotherapy phase I/II trial conducted in primary liver cancer (CheckMate 040), starting in 2012, tested dose escalation and expansion of the PD-1 inhibitor nivolumab involving 262 patients with advanced HCC after first-line treatment with sorafenib. 18 Nivolumab managed to achieve an objective response rate (ORR) of 20% in the final-dose expansion phase as well as a median overall survival (mOS) of 13.2 months with a manageable safety profile. Because of these positive results, a randomized, placebo-controlled phase III trial (CheckMate 459, Table 1 ) compared the effect of nivolumab monotherapy to sorafenib as first-line treatment. 20 The nivolumab treatment arm did not reach its primary endpoint, demonstrating no significant survival benefit compared to sorafenib. Still, nivolumab was discussed by the authors as an option for patients with contraindication(s) to sorafenib, 20 and the favorable safety profile already displayed in CheckMate 040 led to FDA approval of nivolumab as second-line option following sorafenib treatment. 42

**Table 1:** Selected phase III ICI monotherapy trials in advanced or unresectable HCC

Trial	Time of recruitment	Treatment	Line of therapy	Participants	Etiology		Disease stage		Median OS (mo)	Median PFS (mo)	ORR (%)	DCR (%)	Ref.
				*n*	Viral (%)	Nonviral (%)	EHD (%)	BCLC C (%)					
CheckMate 459	01/2016–05/2017	Nivolumab	1st-line HCC	371	54	45	60	82	16.4	3.8	15	55	20
		Sorafenib		372	54	45	56	78	14.7	3.9	7	58	
KEYNOTE-240	05/2016–11/2017	Pembrolizumab + BSC	2nd-line HCC	278	41.4	58.6	70.1	79.9	13.9 ^a^	3.0 ^b^	18.3 ^c^	62.2 ^a^	21
		Placebo + BSC		135	37.1	63	68.9	78.5	10.6	2.8	4.4	53.3	
KEYNOTE-394	05/2017–12/2019	Pembrolizumab + BSC	2nd-line HCC	300	80.4	19.6	77.3	92.3	14.6 ^a^	2.6 ^b^	12.7 ^c^	51	45
		Placebo + BSC		153	81.7	18.3	78.4	95.4	13.0	2.3	1.3	47.1	
RATIONALE-301	12/2017–10/2019	Tislelizumab	1st-line HCC	342	76.1	24	64	79.5	15.9 ^a^	2.1	2.1	44.2	173
		Sorafenib		332	75.8	24.1	59.6	75.9	14.1	3.4	3.4	50.3	

Abbreviations: BCLC, Barcelona Clinic Liver Cancer criteria; BSC, best supportive care; DCR, disease-control rate; EHD, extrahepatic disease; HCC, hepatocellular carcinoma; ICI, immune checkpoint inhibitor; NR, not reached; ORR, objective response rate; OS, overall survival; PFS, progression-free survival, STRIDE, single tremelimumab regular interval durvalumab; TKI, tyrosine kinase inhibitor.

Statistics: one-sided ^a^ < 0.05, ^b^ *p* < 0.01, ^c^ *p* < 0.0001.

Another PD-1 inhibitor, pembrolizumab, was investigated in the phase II KEYNOTE-224 trial. 19 In line with CheckMate 040, the patients were previously treated with sorafenib, and either developed intolerance or showed disease progression. The ORR was 17% (1% complete response, 16% partial responses), the mOS was 12.9 months, and progression-free survival (PFS) was 4.9 months. A follow-up analysis in 2022 even updated the ORR to 18.3% and the mOS to 13.2 months. 43 Following these encouraging results, the randomized, double-blinded phase III trial KEYNOTE-240 ( Table 1 ) tested pembrolizumab as second-line treatment and included 413 patients with advanced HCC who were previously treated with sorafenib in comparison to placebo. 21 Although the trial did not reach statistical significance in 2019, the follow-up in 2020 showed an ORR of 18.3%, a mOS of 13.9 months, and a PFS of 3 months for pembrolizumab (placebo: ORR: 4.4%, mOS: 10.6 months, PFS: 2.8 months). 44 Similar results were shown by KEYNOTE-394 ( Table 1 ), which focused on a primarily Asian cohort. 45 The results were in line with KEYNOTE-240, showing comparable clinical activity and risk profile to other pembrolizumab studies. 19 21 43 44

There have been no large phase III trials investigating ICI monotherapy specifically in CCA. However, the uncontrolled phase II trial KEYNOTE-158 reported a clinical benefit for pembrolizumab monotherapy in pretreated solid tumors with high levels of microsatellite instability. 46 47

In conclusion, ICI monotherapy trials showed an ORR between 12.7 and 18.3% in HCC patients, which is better than standard systemic therapies like sorafenib 6 in first-line and regorafenib 9 in second-line. However, at this point, the ORR and overall survival benefit remain moderate and comparable to the standard therapies.

## Mechanisms of Primary Resistance

One of the main reasons for the moderate efficacy of ICI monotherapy in liver cancer is the relatively high rate of primary therapy resistance—meaning the tumor does not respond to therapy right from the beginning. 48 Primary resistance to immunotherapy strongly depends on cancer type–specific and patient–individual factors, with the composition of the TME playing a major role in shaping therapy response. 49 50 51 52 In brief, an inflamed (or *hot* ) TME shows high infiltration of CTL inside the tumor and the surrounding stroma, alongside high tumor mutational burden (TMB), heightened PD-L1 and interferon (IFN)-γ expression, and has been associated with favorable ICI therapy response. 53 In contrast, a *cold* TME is deserted of any T cell infiltration or PD-L1 expression, and *excluded* TME display CTL and other effector cells gathering on the tumor margin without being able to infiltrate due to stromal barriers and deviant vascular structure. 54 Finally, *immunosuppressed* TME shows moderate T cell infiltration and counteracting immunosuppressive adaptation of the TME such as high expression of interleukin (IL)-10 and transforming growth factor-beta (TGF-β), as well as excessive amounts of T _reg_ , tumor-associated macrophages (TAM), and myeloid-derived suppressor cells (MDSC; Fig. 1 ).

In general, primary resistance mechanisms are categorized into intrinsic and extrinsic mechanisms 55 56 — we will describe these in the following sections, mainly focusing on the TME, as primary resistance in HCC has recently been discussed in detail in *Seminars in Liver Disease* . 57

### Tumor Intrinsic Mechanisms

**Fig. 2:**
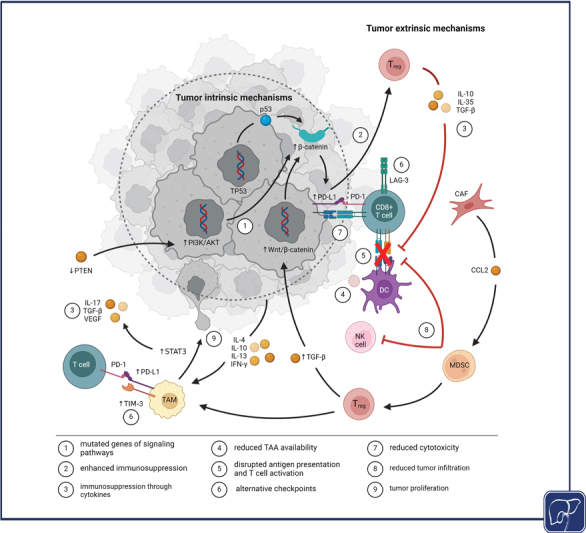
Intrinsic and extrinsic mechanisms of primary resistance. Tumor intrinsic mechanisms are caused by mutations of genes driving resistance-associated signaling pathways (1) that impair function and efficacy of the immune response by enhancing immunosuppressive properties of regulatory T cells (T _reg_ ) (2) through stimulated release of immunosuppressive cytokines (3). Reduced availability of tumor-associated antigens (TAA) (4) causes disrupted antigen presentation resulting in impaired activation of cytotoxic T lymphocytes (CTL) (5). Tumor extrinsic mechanisms involve overexpression of programmed cell death ligand 1 (PD-L1) and alternative checkpoints (6) that reduce cytotoxicity of CTL (7), and recruitment of immunosuppressive cells such as cancer-associated fibroblasts (CAF), myeloid-derived suppressor cells (MDSC), tumor-associated macrophages (TAM), and T _reg_ that prevent tumor infiltration by T cells and NK cells (8). TAM can also promote tumor proliferation (9) and angiogenesis. (Created with biorender.com.) CAF, cancer-associated fibroblasts; CCL, C -C-chemokine; DC, dendritic cell; IFN, interferon; IL, interleukin; MDSC, myeloid-derived suppressor cells; NK, natural killer; PD-(L)1, programmed death (ligand) 1; PTEN, phosphatase and tensin homolog; TAM, tumor-associated macrophage; TGF-β, tumor growth factor-beta; TIM-3, T cell immunoglobulin and mucin domain-containing molecule 3; T _reg_ , regulatory T cell; VEGF, vascular endothelial growth factor.

Since successful immune responses to liver cancer depend heavily on correct priming and activation of T cells by APC, tumor intrinsic reasons for primary resistance mainly involve dysfunctional antigen expression or recognition, often caused by a lack of neoantigens, impaired antigen presentation, and mutations of resistance-associated genes and signaling pathways ( Fig. 2 ).

Neoantigens are TAA that are expressed due to cancer-related mutations and play an important role in T cell activation. 58 A low TMB results in a lower quantity of neoantigens in the TME and therefore a lack of tumor immunogenicity, 59 suggesting that TMB might be a suitable biomarker for predictions of ICI therapy efficacy. 60 61 As such, the TMB showed promise as a predictive biomarker for patients with lung, bladder, and head and neck cancers, 62 but its significance heavily depends on the type of solid tumor, the intratumoral CTL levels, and the abundance of neoantigens, resulting in failure to predict an ICI response in cancers like glioma, prostate cancer, and breast cancer. 63 While the TMB can vary depending on the stage, progression, and subtype of primary cancer, it is known to be relatively low in both HCC and CCA 64 65 and a small case series of HCC patients found no correlation between TMB and ICI response. 66 Interestingly, CCA patients with microsatellite-instability high showed a higher TMB and longer survival after ICI treatment, suggesting that while the TMB should not be used as a sole predictive biomarker its impact should be considered for treatment. 67 In addition, CCA tumors are known to have a predominantly cold phenotype and show downregulation of genes responsible for antigen presentation, 68 which greatly impairs the priming function of APC. 69 Excess lipid accumulation and a subsequent overload of DC in HCC are additional reasons for dysfunctional antigen presentation in liver cancer. 70 71

Mutations of genes in important signal transduction pathways—such as the Wnt/β-catenin pathway, which is altered by mutations in many human cancers 72 and especially prevalent in HCC 73 —can also impact the TME and increase the probability of immune evasion of the tumor contributing to primary resistance. 74 Upregulated β-catenin increases PD-L1 expression with a subsequent reduction of cytotoxicity of CTL, inhibition of DC and T cell recruitment, and enhanced immunosuppression by T _reg_ . 75 In HCC, β-catenin drives transcription of telomerase reverse transcriptase (TERT), which encodes the catalytic subunit of telomerase and is partly responsible for increased tumorigenesis and resistance. 76 Additionally, the canonical Wnt/β-catenin pathway can be stimulated by TGF-β, 77 a mediator that enhances fibrogenesis in the liver and is abundant in an immunosuppressive TME, 78 further promoting tumor proliferation and causing a TME deprived of APC and CTL. 79 In mouse models, β-catenin activation has been shown to promote immune evasion and resistance to anti-PD-1 monotherapy of HCC, 80 which could be overcome by combination therapy (anti-PD-L1 and anti-VEGF). 81 However, data from human trials are conflicting. While several studies found no correlation between these mutations and therapy efficacy, both for atezolizumab and bevacizumab combination therapy 82 and anti-PD-1 monotherapy, 83 the Imbrave150 trial reported a greater survival benefit for patients without mutations of the Wnt/β-catenin pathway treated with atezolizumab/bevacizumab. 81 Further research is needed to explore the dual nature of Wnt/β-catenin mutations, which appear not to be indicative of a universally negative prognosis but rather dependent on the type of applied ICI therapy.

Another driving factor may be mutations of the TP53 gene encoding the p53 transcription factor, a sensor for cellular stress also known as “guardian of the genome”, which are common occurrences in cancer patients. In HCC, TP53 mutations correlate with a noninflamed TME and reduced survival of patients. 84 It has been shown that certain TP53 mutations can induce the Wnt/β-catenin pathway, 85 although the exact interplay of these pathways is still being studied. Additionally, infiltration of T cells in multiple tumors and therefore ICI efficacy can be negatively impacted by loss of phosphatase and tensin homolog (PTEN) and a subsequent activation of the PI3K/AKT pathway. 86 This pathway is dysregulated both in CCA and HCC. 87 88 Other notable mutations in HCC and CCA are of the MYC oncogene and Kirsten rat sarcoma virus (KRAS) gene, which in turn induces overexpression of the MYC oncogene, leading to increased oncogenesis, suppressed immunity and antigen presentation, and activated TGF-β signaling. 89 90 91 92 93 Likewise, isocitrate dehydrogenase 1 (IDH1) mutations are common in CCA 94 and have been linked to immune evasion in mouse models. 95 Finally, signaling pathways like mammalian target of rapamycin (mTOR), 96 TGF-β, ^78^ and IFN-γ/JAK/STAT 97 98 are frequently affected by mutations in primary liver cancer, promoting tumor proliferation and potentially adding to ICI resistance.

### Tumor Extrinsic Mechanisms

Contrary to tumor intrinsic mechanisms, extrinsic mechanisms promote resistance to ICI therapy through cells, cytokines, and metabolites not originating from the tumor. This includes T cell–related events like alternative checkpoint inhibition, T cell exhaustion, recruitment of immunosuppressive cells like T _reg_ , TAM, MDSC, or cancer-associated fibroblasts (CAF), and the effect of immunosuppressive cytokines and chemokines released from tumor cells and immune cells ( Fig. 2 ).

T _reg_ physiologically maintains self-tolerance and immune homeostasis, which is crucial in the liver but creates an avenue for tumors to evade host immunity. Multiple mechanisms exist for T _reg_ to suppress immune responses, 99 for example, through the disruption of DC function or release of immunosuppressive cytokines like IL-10, IL-35, or TGF-β, which downregulate effector T cell functions. 48 TME-resident T _reg_ , as well as TAM, CAF, and MDSC, can prevent tumor infiltration by CTL and NK cells in HCC and CCA 100 101 and high T _reg_ to effector cell ratios are associated with worse clinical outcomes in ICI-treated HCC. 81 Studies have also shown that high TGF-β expression predicts poor prognosis in HCC and CCA, 102 103 104 thus potentially promoting primary resistance to ICI therapy.

MDSC are immunosuppressive cells that stem from immature myeloid cells whose differentiation into granulocytes, macrophages, or DC is prevented by the TME. MDSC are present only in patients with pathological conditions like cancer, and they promote angiogenesis and metastasis of tumors while showing immunosuppressive effects upon activation. 105 They can interrupt the cell cycle of T cells and trigger cell death via apoptosis. 106 MDSC can also influence the antigen presentation of DC and increase the proliferation of T _reg_ , promoting tumor growth in HCC 107 and CCA. 108 In CCA, CAF recruit MDSC to the TME via C-C chemokine ligand 2 (CCL2), contributing to tumor growth and cancer stemness, which impacts the resistance of the tumor to cancer therapy. 109 110 In this context, treatment-induced immunosuppression can be further aggravated through the IL-1β-dependent recruitment of suppressive myeloid populations and the disruption of CD8 T cell responses. 111 In both HCC and CCA, host genetic variations in TME-modulating mediators like IL-1β and the IL-8 pathway have been associated with both oncological and overall prognosis. 112 113 114

TAM are comprised of different subtypes that are either proinflammatory or anti-inflammatory with tissue-restorative functions. 115 They display a high plasticity and can be repolarized by the surrounding environment to serve the needs of the immune system. Tumors often take advantage of the pro-regenerative properties of TAM, which can be induced by cytokines such as IL-10, IL-4, and IL-13, 116 to promote angiogenesis, tumor proliferation, and metastasis. 117 118 Polarization of TAM is also influenced by tumor-secreted molecules like the sonic hedgehog protein or succinate and by immune cells like T _reg_ or MDSC through the release of IFN-γ or downregulation of the signal transducer and activator of transcription 3 (STAT3) pathway. 119 In HCC, strong expression of PD-L1 on TAM and a consequential suppression of the T cell response have been shown to result in poor prognosis for patients. 107 120 This may promote resistance of tumors to ICI therapy. Infiltration of TAM has been associated with disease progression in CCA patients as well due to activation of STAT3, and subsequent tumor proliferation. 121 Activation of STAT3 also causes the production of immunosuppressive cytokines like TGF-β, IL-17, and vascular endothelial growth factor (VEGF), contributing to ICI resistance. 122 123 The immunosuppressive properties of TAM and their role in resistance to checkpoint therapy make them a possible target to overcome ICI resistance. 124

CAF are abundant in the tumor stroma and are involved in remodeling of the extracellular matrix in the TME, thus playing an essential role in primary liver cancer as HCC often arises from a fibrotic, premalignant microenvironment while in highly desmoplastic CCA, the fibrous tissue develops in parallel with the tumor. 125 Mainly originating from activated hepatic stellate cells (HSC), 126 they promote tumor cell proliferation and orchestrate an immunosuppressive TME. 127 128 Myofibroblastic HSC and CAF promote disease progression and tumorigenesis in both CCA and HCC. 129 130 Interestingly, subpopulation analysis also revealed HSC with tumor-suppressing characteristics during hepatocarcinogenesis. 130

In addition to immunosuppressive cells and cytokines produced in the TME, alternative inhibitory immune checkpoints like TIM-3 and LAG-3 can promote resistance to ICI therapy. 131 TIM-3 was found to be upregulated on TAM in the TME due to TGF-β exposure, 132 suggesting an alternative route of checkpoint inhibition used by cancer cells that would not be covered by PD-1/PD-L1 or CTLA-4 ICI therapy. Coexpression of LAG-3 and PD-1 on tumor-infiltrating lymphocytes results in a cooperative immunosuppressive effect 133 that could be upheld to some degree even after ICI monotherapy targeting PD-1/PD-L1 resulting in resistance. As LAG-3 and TIM-3 are upregulated and related to poor prognosis in HCC patients, it is relevant to consider this cause of resistance to ICI therapies. 134 135

### Liver-Specific Mechanisms

The unique symbiosis of the liver with the gut and its microbiome, also referred to as the gut–liver axis, requires an intricate balance of immune surveillance and self-tolerance in the liver. 136 It is constantly exposed to diverse microorganisms, antigens, and microbial products through the portal vein, which demands complex regulation of the immune system to eliminate pathogens while avoiding autoimmune responses. 137 Consequently, macrophages in the (healthy) liver, particularly liver-resident Kupffer cells, are equipped to support immunosuppression. 138 Similarly, DC located in the liver have a lower expression of co-stimulatory molecules and thus a lower capacity to activate T cells. 137 139 Liver tumors can use this to evade the immune system and limit the effect of potential immunotherapy treatments, ultimately causing resistance to ICI.

Most cases of primary liver cancer evolve due to an underlying liver disease—HCC most often on the background of fibrosis and cirrhosis caused by viral hepatitis, alcohol abuse or metabolic liver disease, 140 141 and CCA due to inflammatory processes like primary sclerosing cholangitis (PSC) or parasites. 142 Notably, metabolic dysfunction-associated steatotic liver disease (MASLD) becomes more and more important, as it affects up to 38% of the global adult population. 143 MASLD can range from simple steatosis to MASH (metabolic dysfunction-associated steatohepatitis) with its characteristic hepatic inflammation, which may progress to fibrosis or cirrhosis and is tightly linked with obesity, type 2 diabetes, hypertension, and other cardiovascular diseases. 144 145 Especially severe fibrosis embodies a high-risk factor for the development of primary liver tumors, at least in part due to the activation of fibroblasts with tumor-promoting function in both HCC and CCA. 125 The distinct adaptations of the hepatic immune microenvironment in viral versus non-viral etiologies 146 may also affect the response to ICI therapy and emerging evidence suggests that non-viral HCC may be accompanied by reduced ICI therapy efficacy. 147 148 149 Mechanistically, we and other groups have demonstrated fundamental changes in the hepatic immune microenvironment of both lymphoid 150 151 152 153 and myeloid compartments 154 155 156 in MASLD mouse models and patients. 130 157 158 As commonly available ICIs focus on amplifying CTL function, 159 lymphocytes are a central spotlight in this context. The accumulation of tissue-resident memory T cells as well as autoaggressive exhausted CTL have been shown to drive inflammation and fibrosis in the livers of preclinical MASH models as well as MASH patients. 150 151 Indeed, therapeutic anti-PD1 treatment led to the expansion of these exhausted CTL in the tumors of MASH-HCC-bearing mice but failed to ensure tumor control. Strikingly, prophylactic treatment increased HCC incidence, highlighting the important protective mechanisms of inhibitory checkpoints. 160 Even in PD-1 responsive liver cancer mouse models, efficacy was abrogated by diet-induced MASLD/MASH, which was caused by diet-associated impaired CTL metabolism and motility. Remarkably, this effect could be rescued by additional metformin treatment. 161 CD4+ T cells also play a role in ICI efficacy 162 as evidenced by the MASH-inducing methionine-deficient diet, which leads to loss of hepatic CD4+ T cells aggravating HCC development. 153 Fittingly, subgroup-specific meta-analyses of multiple randomized controlled trials demonstrated a higher survival benefit for patients with viral HCC compared to non-viral etiology. 149 160 However, the matter is more complicated, as patients of non-viral etiology actually seem to benefit from double ICI therapy (anti-PD-L1 plus anti-CTLA-4). 163 Furthermore, it is not clear how many patients with non-viral HCC actually suffer from MASLD. Nevertheless, the impact of the underlying liver disease on the response to ICI therapy is still plausible, and further research is necessary to illuminate on this matter. 164 165

MASLD is tightly connected to obesity and changes in the microbiome, which might also influence response to ICI. Obesity has already been linked to limited tumor control due to leptin-dependent T cell exhaustion in a genetic obesity mouse model 166 and tumor cells of mice fed with a high-fat diet increased lipid uptake, which was followed by metabolic reprogramming and impaired CTL function. 167 Furthermore, metabolic activation of B cells also leads to tumor-promoting dysfunctional T cell responses in this context. 152 Interestingly, leptin-dependent T cell exhaustion increased response to anti-PD-1 therapy 166 and this effect can also be seen in patients with non–small-cell lung cancer (NSCLC) and melanoma. 168 Microbial dysbiosis has also been reported in patients not responding to ICI for many types of cancers including HCC and CCA, 169 170 which leads to immunosuppressive rather than antitumor immune responses. In addition, microbial diversity declined in non-responders over the course of treatment, 169 and antibiotic treatment was associated with shorter survival times. 171

## “Ready-to-Use” Combination Therapy to Overcome Primary Resistance

As described earlier, single-agent PD-(L)1 inhibitor therapies generate lasting antitumor response in subgroups of patients with advanced HCC 18 19 172 but do not demonstrate a significant survival benefit for the overall treatment population compared with tyrosine kinase inhibitors. 20 173 Combining ICI with other, already existing antitumor agents for primary liver cancer represents an accessible choice to overcome primary resistance to ICI therapy ( Table 2 ).

**Table 2:** Selected phase III ICI combination therapy trials in advanced or unresectable HCC and CCA

Trial	Time of recruitment	Treatment	Line of therapy	Participants	Etiology		Disease stage		Median OS (mo)	Median PFS (mo)	ORR (%)	DCR (%)	Ref.
				*n*	Viral (%)	Nonviral (%)	EHD (%)	BCLC C (%)					
*ICI plus ICI*													
HIMALAYA (NCT03298451)	10/2017–06/2019	STRIDE (durvalumab plus tremelimumab)	1st-line HCC	393	59	41	53.2	80.4	16.43 ^e^ (sorafenib)	3.78	20.1	60.1	185
		Durvalumab		389	58.1	41.9	54.5	79.4	16.56	3.65	17.0	54.8	
		Sorafenib		389	57.3	42.7	52.2	83.0	13.77	4.07	5.1	60.7	
*ICI plus MKI (or anti-VEGF antibodies)*													
IMBrave150 (NCT03434379)	03/2018–01/2019	Atezolizumab plus bevacizumab	1st-line HCC	336	70	30	63	82	NR ^f^	6.8 ^f^	27.3 ^f^	73.6	199
		Sorafenib		165	68	32	56	81	13.2	4.3	11.9	55.3	
COSMIC-312 (NCT03755791)	12/2018–08/2020	Atezolizumab plus cabozantinib	1st-line HCC	432	60	39	54	68	15.4	*6.8* ^e^	11	78	203
		Sorafenib		217	60	40	56	67	15.5	*4.2*	4	65	
		Cabozantinib		188	73	37	54	65	n.a.	n.a.	6	84	
ORIENT-32 (NCT03794440)	02/2019–01/2020	Sintilimab + IBI305 (bevacizumab biosimilar)	1st-line HCC	380	96	4	73	85	NR ^g^	4.6 ^g^	21% ^g^	72	201
		Sorafenib		191	98	2	75	86	10.4	2.8	4%	64	
LEAP-002 (NCT03713593)	01/2019–04/2020	Pembrolizumab plus lenvatinib	1st-line HCC	395	63	37	63	78	21.2 ^a^	8.2	26.1	81.3	204
		Placebo plus lenvatinib		399	61	39	61	76	19.0	8.1	17.5	78.4	
CARES-310 (NCT03764293)	06/2019–03/2021	Camrelizumab plus rivoceranib	1st-line HCC	272	84	15	64	86	22.1 ^c^	5.6 ^c^	25 ^c^	78	205
		Sorafenib		271	84	17	66	85	15.2	3.7	6	54	
*ICI plus chemotherapy*													
TOPAZ-1 (NCT03875325)	02/2019–12/2020	Durvalumab plus gemcitabine and cisplatin	1st-line CCA	341	n.a.		55.7 (iCCA)	88.9 (metastases)	12.8 ^d^	7.2 ^f^	26.7	85.3	221
		Placebo plus gemcitabine/cisplatin		344			56.1 (iCCA)	83.1 (metastases)	11.5	5.7	18.7	82.6	
KEYNOTE-966 (NCT04003636)	10/2019–06/2021	Pembrolizumab plus gemcitabine/cisplatin	1st-line CCA	533		60 (iCCA)	89 (metastases)	12.7 ^b^	6.5 ^a^	29	75	223
						Placebo plus gemcitabine/cisplatin		536	n.a.		58 (iCCA)		88 (metastases)	10.9	5.6	29	76	

Abbreviations: BCLC, Barcelona Clinic Liver Cancer criteria; CCA, cholangiocarcinoma; EHD, extrahepatic disease; DCR, disease-control rate; HCC, HCC, hepatocellular carcinoma; ICI, immune checkpoint inhibitor; n.a., not applicable; NR, not reached; ORR, objective response rate; OS, overall survival; PFS, progression-free survival, STRIDE, single tremelimumab regular interval durvalumab; MKI, multikinase inhibitor.

Statistics: one-sided ^a^ *p* < 0.05, ^b^ *p* < 0.01, ^c^ *p* < 0.0001; two-sided ^d^ *p* < 0.05, ^e^ *p* < 0.01, ^f^ *p* < 0.001, ^g^ *p* < 0.0001.

### Combination of Immune Checkpoint Inhibitors

To further accelerate antigen-mediated CTL cytotoxicity, blocking additional inhibitory checkpoint molecules and stimulating activating ones 174 are standard strategies regarding combination therapy. The most commonly combined immune checkpoint antibodies in liver cancer are anti-PD-L1 and anti-CTLA-4. The rationale behind this combination is that blockage of the PD-1/PD-L1 pathway does not necessarily lead to antitumor immunity, if PD-1+ CTLs are not present in the tumor. 34 Furthermore, in case they are present, their actions might be counteracted by immunosuppressive T cells such as T _reg_ . For instance, single anti-PD-1 treatment resulted in expansion of exhausted CTL (PD-1 + , LAG-3 + , TIGIT + ) but failed to induce tumor control in a HCC mouse model. 175 Furthermore, CTLA-4 blockade increases activation of CTL in the lymph node and therefore the probability of cancer antigen–specific CTL infiltrating the TME 34 ( Fig. 1 ). The synergistic effects of anti-CTLA—direct enhancement of effector T cell function as well as inhibition of immunosuppressive T _reg_ and indirect higher probability of DC maturation during the priming phase 34 174 176 — in conjunction with anti-PD-1/PD-L1 blockade in the immune effector phase have been verified as a therapeutic option in various advanced cancers including melanoma, colon, and lung cancer. 177 178 179 180

First results on anti-PD-1/CTLA-4 combination therapy originated from the randomized phase I/II trial CheckMate 040, testing safety and efficacy of three distinct nivolumab plus ipilimumab sequences in a HCC patient cohort previously treated with sorafenib. 18 All study arms showed promising ORR of approximately 30%, with the highest complete response rates and mOS (22.8 months) observed for patients receiving four doses of 1 mg/kg nivolumab plus 3 mg/kg ipilimumab for every 3 weeks followed by 240 mg nivolumab every 2 weeks (study arm A). 181 At this time, approved second-line MKI therapy demonstrated only a maximum of 10.6 months of mOS, 9 10 11 resulting in accelerated approval of nivolumab combined with ipilimumab for second-line advanced HCC therapy in the United States. 182 The improvement of ORR and OS may directly correlate to increasing dosages of ipilimumab, which coincides with higher rates of adverse effects. Nevertheless, the benefit–risk profile still favors the combination therapy with higher anti-CTLA-4 dosage. 183 In another phase I/II trial, the STRIDE (single-dose tremelimumab [anti-CTLA-4] with regular interval durvalumab [anti-PD-L1]) regime demonstrated highest ORR (24%), mOS of 18.7 months, and most prominent increase of proliferating peripheral CTL 2 weeks after starting therapy in advanced HCC patients. 184 The following randomized, controlled phase III trial (HIMALAYA), which tested STRIDE and durvalumab monotherapy against standard of care sorafenib in treatment-naive advanced HCC, demonstrated superior OS of STRIDE against sorafenib (16.43 vs. 13.77 months) and noninferiority of durvalumab monotherapy to sorafenib. 185 Following this trial, STRIDE received FDA approval and was included as a first-line option for advanced HCC in clinical practice guidelines. 186 187 188

In noncontrolled phase I/II studies on pretreated CCA, the combination of nivolumab/ipilimumab surprisingly did not cause superior ORR and mOS (23 and 5.7 months, respectively) 189 to nivolumab monotherapy (22 and 14.2 months). 190 This may have resulted from differences in study ORR assessment and patient exclusion criteria 189 as well as relatively low doses of ipilimumab (1 vs. 3 mg/kg in pretreated HCC 181 ). In another phase II study including Asian patients with advanced and pretreated CCA, combination therapy of durvalumab and tremelimumab in comparison to durvalumab monotherapy managed to obtain moderately higher ORR (10.8 vs. 4.8%) and a comparable safety profile. 191 Further studies are required to access the leverage of combined ICI therapy in CCA.

### Combining ICI and Targeted Therapy (TKI or Antiangiogenic Drugs)

Tumor angiogenesis is one of the essential hallmarks of cancer. 192 Hypoxia during tumor growth triggers expression of proangiogenic factors such as VEGF by upregulation of hypoxia-inducible factor proteins and causing neoangiogenesis. 193 Additionally, VEGF facilitates essential immunosuppressive functions by undermining leukocytes–endothelial cell adhesion and DC maturation, impairing CTL proliferation and function by promoting their exhaustion and increases T _reg_ infiltration. 194 Normalizing this VEGF-suppressed TME by using inhibitors of VEGF (antibodies, e.g., bevacizumab or VEGFR-targeting MKIs, e.g., sorafenib or lenvatinib) might synergize with anti-PD-1/PD-L1 therapy to more effectively unleash CTL-mediated cancer cell killing. 195 Another advantage of this combination therapy might be that ICI counteracts intratumoral PD-L1 upregulation caused by antiangiogenic therapy. 196 Anti-VEGF therapy itself might therefore create an even more immunosuppressive TME, which could be unleashed by ICI therapy. Combinations of antiangiogenic and ICI therapy have already been successfully introduced in other solid cancers such as renal cell carcinoma. 197 198

Combination of the PD-L1 inhibitor atezolizumab with bevacizumab demonstrated longer PFS than atezolizumab alone in a phase Ib study with previously untreated advanced HCC patients. 172 Its successor, the randomized phase III trial, IMbrave150 demonstrated significantly reduced risk to die in the patient groups treated with combination therapy, with significantly longer PFS, higher ORR, and 20% more disease control until the time of first analysis. 199 The significant survival benefit of anti-PD-L1 plus anti-VEGF therapy was confirmed in the updated data analysis from 2022 200 and with the China-based ORIENT-32 phase III trial, which also reported significantly prolonged OS and PFS when treated with anti-PD-L1 sintilimab and IBI305 (bevacizumab biosimilar). 201 In addition, the randomized phase III IMbrave050 trial demonstrated that adjuvant treatment with the atezolizumab/bevacizumab combination after resection or ablation improved recurrence-free survival versus active surveillance, 202 further highlighting the growing impact of ICI in curative treatment settings.

Because ICI combination with anti-VEGF antibodies was successful, it seemed evident to combine ICI with TKI, as they already have a significant beneficial effect on their own and block more pathways than VEGFR1/2 alone. Surprisingly, atezolizumab in combination with cabozantinib (COSMIC-312), although demonstrating significantly prolonged PFS, failed to improve OS and caused more treatment-associated adverse effects. 203 Along the same lines, the combination of pembrolizumab and lenvatinib in LEAP-002 failed to meet the prespecified boundaries for superiority in both OS and PFS. 204 The first trial showing significant advantages for the combination of ICI and TKI over TKI alone for both OS and PFS was the randomized, open-label phase III CARES-310 trial, comparing dual therapy of anti-PD-1 antibody camrelizumab with VEGFR2-targeting TKI rivoceranib (also known as apatinib) with sorafenib alone. 205 Here, combination therapy significantly prolonged mOS (22.1 vs. 15.2 months) and PFS (5.6 vs. 3.7 months). The ORR for the combination therapy was 25% and the disease control rate was 78% (vs. 54%). Just recently, combination of nivolumab and regorafenib demonstrated an impressive 1-year survival of 80.5% and ORR of 30.5% in a multicenter, single-arm phase II study including treatment-naive patients with advanced HCC, 206 clearing the way for possible future first-line treatments.

Although the efficacy of single-agent antiangiogenic 207 or ICI therapy in CCA remains limited, their combination created some encouraging results. For example, in a phase II trial, which included 32 patients with advanced CCA, treatment with pembrolizumab and lenvatinib in second-line resulted in an ORR of 25% and mOS of 11 months, which exceeded the results from monotherapy trials. 208

### Combination of ICI and Chemotherapy

Conventional chemotherapy is generally not recommended for HCC treatment, as HCC is resistant to the most common regimes and chemotherapy may aggravate underlying cirrhosis, leading to inconclusive or even negative survival benefits. 209 In comparison, Gem/Cis has been the standard first-line chemotherapy for patients with advanced CCA since the ABC-02 trial in 2010. 7

While platinum-based chemotherapeutics mainly take effect by inducing apoptosis due to DNA strand breaks, 210 they have immunostimulatory properties as well. 211 212 Cisplatin has been shown to increase the expression of MHC class I antigens on cancer cells and tumor-associated DC, recruit effector cells to the TME, increase cytolytic activity of CTL, and reduce the infiltration of immunosuppressive cells. 211 Additionally, gemcitabine may reduce the number of MDSC in tumor-bearing mice. 213 Since the TME of CCA has been described as mainly immunosuppressive with high infiltration of immunosuppressive cells or immune-exclusive with low T cell infiltration and low major histocompatibility complex (MHC)-I/PD-L1 expression, 214 the combination of immune checkpoint inhibition with Gem/Cis or other approved chemotherapeutics has been recognized as a successful antitumor concept. Furthermore, the concept was already proven in other advanced tumors such as NSCLC 215 and triple-negative breast cancer. 216

Therefore, different combinations of ICI and chemotherapy have been evaluated in early-phase clinical trials throughout the last years. 217 218 219 220 The most promising one tested three different sequences of Gem/Cis plus durvalumab alone or with tremelimumab. Notably, patients with immediate combination of Gem/Cis with durvalumab alone or durvalumab plus tremelimumab displayed high objective responses of approximately 70%. 220 While adding tremelimumab to the regimen achieved no additional benefit in ORR and OS, the combination of durvalumab and Gem/Cis has been further explored in the phase III TOPAZ-1 trial, 221 which tested the anti-PD-L1 drug durvalumab with chemotherapy ( *n* = 341) compared to placebo with chemotherapy ( *n* = 344) in patients with advanced CCA. The OS over 24 months was 24.9% for the durvalumab cohort and 10.4% for the placebo cohort, with an ORR of 26.7 and 18.7%, respectively. The median PFS for durvalumab was 7.2 months compared to 5.7 months in patients treated with placebo. The achieved results and safety profiles were comparable with above-mentioned ICI monotherapy trials in HCC. Because of these encouraging results, TOPAZ-1 became the new standard therapy in advanced CCA. 222 Results from KEYNOTE-966, a phase III trial testing the combination of pembrolizumab and Gem/Cis in first-line setting in CCA, were similarly encouraging. 223 Consequently, combining ICIs with chemotherapy became standard of care for advanced CCA. 224

## Mechanisms of Acquired (Secondary) Resistance

Tumors that initially respond to ICI therapy often develop acquired or secondary resistance through adaptive mechanisms. 225 Considering the fact that due to availability of advanced combinations more liver cancer patients are responding to ICI therapy, it can be expected that a substantial amount of patients will also have to deal with acquired resistance. 48 As ICI combination therapies have only been used in standard settings so far, rates of acquired resistance have not yet been widely reported for liver cancer. In other tumor types, acquired resistance has been shown to affect up to 65% of patients in up to 4 years of follow-up. 225

The specific mechanisms driving acquired resistance are in many aspects still elusive—especially regarding primary liver cancer—but are to some degree overlapping with components of primary resistance. 226 Collected clinical reports suggest reduced TAA availability, disrupted antigen presentation, altered IFN-γ signaling, promotion of an excluded/immunosuppressive TME, and upregulation of (alternative) checkpoints as possible mechanisms. 225 In addition, emerging data suggest that a considerable proportion of patients develop anti-drug antibodies against certain ICI. 227 For example, 28% of tested patients developed antibodies against atezolizumab during IMbrave150, 146 while less than 5% of patients developed antibodies against durvalumab or tremelimumab during the HIMALAYA trial. 185 While the impact of these drug-neutralizing antibodies on primary and secondary resistance is not completely understood, they might interfere with the treatment efficacy, as patients who develop antibodies against atezolizumab early during therapy are less likely to benefit. 146 228

These mechanisms are often driven by escape mutations. For example, tumors can lose the encoding sequences for key TAA related to the initial ICI response by sub-clone elimination or genomic alterations. 229 230 In consequence, expanding adapted tumor clones are more protected from antigen-associated CTL killing. Furthermore, new mutations can favor a less immunogenic TME. Alterations of beta-2-microglobulin (B2M) lead to reduced or disturbed MHC class I expression on tumor cells, which hinders effective antigen recognition by APC, and are common findings in lung cancer and melanoma patients with acquired resistance. 231 232 233 In line with this, mutations in Janus kinase 1 and 2 (JAK1/2) disrupt the IFN-γ (released from effector T cells) signaling pathway in tumor cells, reducing the expression of MHC class I and PD-L1 and thereby hindering tumor cell killing. 234 235 Another mechanism for acquired resistance is the immunosuppressive reshaping of the TME by immunosuppressive cytokines. Loss of tumor suppressor PTEN or activity of the Wnt/β-catenin pathway, which are also linked to primary resistance (see above), can lead to immunosuppressive cytokine production and defective DC priming. 86 236 Upregulation of alternative immune checkpoints such as TIM-3 or LAG-3 contributes to T cell exhaustion 237 238 and their expression on (potential) APC can negatively influence the antigen presentation mechanisms. 239 240 241

Since data on acquired resistance are still sparse in liver cancer, the question remains whether (and how) to treat patients after acquired therapy failure. For this, a distinct analysis and knowledge of the therapy-altered TME is likely to be key. In clinical practice, re-challenge with a different regimen of ICI after failure of first-line ICI therapy in HCC is oftentimes considered and demonstrated (some) efficacy. 242 Alternatively, novel combination therapies with the possibility to completely eliminate the tumor might be able to prevent acquired resistance altogether.

## The Future of ICI Therapy—How to Heat up the TME

As discussed in the previous paragraphs, single ICI therapy most likely shows benefit for patients when the tumor has a *hot* TME. As the majority of primary liver cancer displays an immune-negative TME, 214 243 which can be caused by various altered and dysregulated processes during the cancer-immunity cycle, 244 altering these hallmark steps and reshaping the TME using additional agents might open a window of opportunities for ICI to work its magic. 52 245 246 Recently, established double-combination therapies have already shown promising effects (see above), but current research suggests to explore even more complex therapy regimes. Here, we focus on the most prominent developments and provide an overview on preclinically and clinically explored therapeutic options ( Fig. 1 ). However, as a note of caution, HCC — and to some extent also CCA — develop in a chronically injured, oftentimes fibrotic or cirrhotic liver; any attempt to reinforce inflammation (to “heat up” the TME) needs to consider the risk of aggravating liver inflammation and fibrosis, that is, aggravating the underlying liver disease. 115

### Inducing Immunogenic Cell Death

Immunologically cold tumors are characterized by a lack of TAA and APC-recruiting danger signals and therefore show absence of overall immune cell infiltration, which in turn makes beneficial ICI therapy highly unlikely. 50 While the main goal of established primary liver cancer treatment options, such as chemotherapeutics, targeted therapy, and local treatment, is the reduction of tumor burden, they also have been shown to stimulate tumor-specific immune responses by immunogenic cell death (ICD). 247 ICD is a form of regulated cell death, which triggers adaptive immune responses in the host and is characterized by simultaneous release of TAA as well as immunostimulatory DAMP (such as heat shock proteins, high-mobility-group protein B1, and adenosine triphosphate), which recruit DC and other APC to the tumor. 248

The possibilities of radiation therapy include SBRT (stereotactic body radiation), SIRT (selective internal radiotherapy), and transarterial radioembolization. While liver toxicity is a limiting factor especially for patients with underlying liver disease, local application is a substantial option for patients with primary liver cancer. 4 Apart from the induced DNA damage to the tumor cells, which is the primary effect, radiation has been shown to improve TAA and DAMP release as well as type 1 IFN production, followed by DC infiltration and maturation and increased CTL infiltration. 249 Furthermore, radiation can facilitate the upregulation of MHC class I expression. 250 Some encouraging results have already been detected for the combination of nivolumab/SIRT and nivolumab/ipilimumab/SBRT in HCC. 251 252 Notably, radiation can also have immunosuppressive effects on the TME, which we will discuss later.

Conventional chemotherapy, especially platin-based therapy regularly used in CCA treatment, can be an effective inducer of ICD as well. 253 In HCC, local chemotherapy application to the tumor via transarterial chemoembolization (TACE) remains a standard treatment option for intermediate stages and has been shown to upregulate proinflammatory pathways. 254 In this regard, the EMERALD-1 phase III trial recently demonstrated significantly prolonged PFS when TACE-eligible patients with unresectable HCC were additionally treated with durvalumab and bevacizumab, possibly laying the foundation for improved standard treatments of advanced HCC. 255 Other locoregional therapies—such as radio frequency or thermal ablation—also induce ICD characterized by the release of DAMPs like heat shock proteins, 256 257 and combination therapy with ICI is being investigated in great depth in this regard. 254

Oncolytic viruses (OVs) are another approach to induce ICD, engineered to exclusively infect and lyse tumor cells. 258 While there is, up to this point only, preclinical evidence for successful OV application in CCA, they have been used in clinical HCC trials. 259 The most prominent, JX-594 (commercially: Pexa-Vac), an oncolytic pox virus vaccine additionally expressing GM-CSF and β-galactosidase for APC recruitment, was shown to induce antibody-mediated cancer cell cytotoxicity and TC activation in a rabbit VX2 tumor model as well as in humans with various solid tumors. 260 While it also demonstrated dose-dependent improved survival in a dose-finding trial, 261 JX-594 did not improve survival as second-line therapy after HCC patients progressed on sorafenib, although increased TC responses were observed in the therapy arm. 262 Nevertheless, a combination with nivolumab to amplify ICI treatment efficacy is currently under investigation (NCT03071094).

### APC Recruitment, Activation, and TC Priming

Following the release of TAA and DAMP, the recruitment and activation of APC are the next essential steps in the cancer immunity cycle. Cytokines, such as FMS-like tyrosine kinase 3 ligand (Flt3L), are able to attract DC, which can subsequently be activated by adjuvants, like toll-like receptor (TLR) agonists. 263 PolyIC, a synthetic TLR3 agonist, induces activation and maturation of conventional DC type 1 264 and is currently tested in combination with anti-PD-1 in HCC patients (NCT03732547). The TLR9 agonist CpG 265 has not yet been explored in clinical HCC, but intratumoral injection of CpG into orthotopic and ectopic HCC mouse models together with anti-OX40 significantly slowed down tumor growth and inhibited T _reg_ and MDSC infiltration to the tumor site while increasing CTL infiltration. 266 The CD40 receptor, which is expressed on activated APC, drives activation of CTL by engaging with its ligand CD40L, leading to optimized priming with clonal T cell expansion and CTL infiltration when combined with chemotherapy. 267 The combination of anti-PD1, CD40 agonist and gemcitabine/cisplatin chemotherapy, has been shown to significantly improve survival when compared with chemotherapy treatment alone in preclinical CCA models. 268 A similar combination is currently explored in a Phase 1/2 trial (NCT05849480).

### Boost Infiltration of Immune Cells

In an excluded TME, immune cells linger at the border of the tumor and do not enter the tumor stroma, which is further exacerbated by reduced pH, hypoxia, and nutrient availability. 49 Treatment against VEGF, with antibodies or MKI, as well as TACE have shown to positively influence the chaotic angiogenesis of the TME, which helps immune cell infiltration. 269 In addition to other immune effects, yttrium-90 radioembolization has been shown to upregulate the chemokine C-C motif ligand 5 (CCL5) and chemokine C-X-C motif ligand 16 (CXCL16) pathway in the tumors of HCC patients, which led to boosted CTL and NK cell infiltration. 270

The formation of a tumor immune barrier, containing CAF and secreted phosphoprotein 1 (SPP1)+ macrophages, at the tumor border of HCC patients correlates with reduced response to anti-PD-1 therapy 271 and, therefore, stroma-modifying therapy is an interesting approach for combination therapy. While the data on CAF therapy in liver cancer is still sparse, targeting CAF-specific proteins like fibroblast activation protein, repolarization of the myofibroblastic phenotype, and targeting of CAF-derived signals seems promising. 272 For example, combination of a CXCR4 inhibitor (receptor for CAF-produced CXCL12) and anti-PD-L1 reduced tumor growth in a mouse pancreatic cancer model, 273 and combination of TGF-β blockade and PD-L1 antibody therapy increased T cell infiltration into the tumors of immune-excluded mouse breast cancer models. 274

### Counteracting Immunosuppressive Adaptations

MKIs are well established as first- and second-line therapy options for patients with advanced HCC. 209 In a substantial proportion of selected patients with CCA, molecularly targeted therapy may be an option, with several approved drugs either targeting oncogenic fibroblast growth factor receptor 2 (FGFR2) fusions or gain-of-function variants of IDH1. 275 Their influence on the VEGFR pathway is thought to increase influx of T cells to the TME and patients with HCC already greatly benefit from the VEGF(R)-targeting therapy and ICI combination ( Table 2 ). Nevertheless, preclinical HCC data also suggest immunosuppressive effects. Sorafenib promotes immunosuppression by PD-L1 upregulation and CXCR4- mediated infiltration of T _reg_ and M2-polarized macrophages. 276 In this case, treatment with anti-PD1 showed only additional effects when combined with sorafenib and anti-CXCR4 but not sorafenib alone, making a case for targeting immunosuppressive pathways and cells as an important feature of combination immunotherapy. 276 Furthermore, radiation therapy can also shape the TME in an immunosuppressive way. 263 For example, post-TACE TME analysis of HCC patients showed reduced numbers of infiltrating CTL and increase triggering receptor expressed on myeloid cells (TREM)2+ TAM compared to pre-TACE TME. 277 Therefore, if ICD is induced, immunosuppressive counterregulation might be a consequence. For example, the Flt3L-dependent infiltration of DC to the TME may be accompanied by T _reg_ influx. 278

Hence, the importance of counteracting immunosuppressive cells has been widely discussed and future combination therapies have to address these issues. Combinations of anti-PD1 antibodies with anti-CTLA-4 34 or VEGF-inhibitors 195 have been shown to counteract T _reg_ and combining doxorubicin (commonly used in TACE) with a mitophagy-inducing drug also reshaped the TME towards more memory and effector T cells and less T _reg_ in a mouse model of HCC. 279

Blocking immunosuppressive myeloid cells has also been explored in the field. An interesting preclinical example has been recently published: in an HCC mouse model, ferroptosis induction resulted in increased CTL infiltration which was counteracted by tumor cell PD-L1 upregulation. While combination with anti-PD-1 therapy did result in a modest survival benefit, only the combination of ferroptosis induction, anti-PD1, and blockade of C-X-C-chemokine receptor 2 (CXCR2)-mediated MDSC infiltration led to a long-lasting antitumor response. 280 Also, the combination of anti-CXCR2 and anti-PD-1 was shown to be very effective in a preclinical model of steatosis-induced HCC. 281 This combination is currently under clinical investigation in HCC. 282 Colony-stimulating factor 1 (CSF1) blockade also represents a new combination target currently approached in liver cancer therapy (NCT05438420)—it has been shown to prevent migration and activation of TAM and enhanced antitumor immunity when combined with anti-PD-L1 in an osteopontin-overexpressing HCC mouse model. 283 Similarly, PD-L1 expressing TAM in concert with MDSC facilitated tumor growth in a murine CCA model and only inhibition of TAM infiltration (via CSF1R blockade) combined with MDSC depletion resulted in response to PD-1 blockade. 284

TGF-β is one of the most prominent cytokines to promote tumor growth and immunosuppressive functions in the TME, 285 and TGF-β inhibitors have been hypothesized to make the TME more approachable for other immunotherapies. Bintrafusp alfa, a bifunctional fusion protein combining anti-PD-L1 with a “TGF-β-trap,” has shown some favorable results in second-line setting 286 for CCA. In contrast, a number of clinical trials with TGF-β inhibitor and ICI combination failed to show enhanced antitumor immunity — the combination with ICD inducers might be essential for this therapy to work. 287

The expression of alternative checkpoints such as TIM-3 and LAG-3 also contributes to an immunosuppressive TME. 225 Recently, combination of nivolumab and relatlimab (anti-LAG-3) demonstrated favorable PFS compared to nivolumab monotherapy in patients with advanced treatment-naive melanoma in a phase III trial. 288 Compared to this, encouraging trial data on anti-TIM-3 are limited. First results on TIM-3 antibody monotherapy in a phase I trial in advanced solid cancers showed only little response. 289 In advanced MSI-H/dMMR tumors (phase Ib), monotherapy as well as combination with anti-PD-(L)1 showed surprisingly high response rates up to 45%. 290 Of note, responses were always more favorable if patients initially responded to anti-PD-(L)1 therapy, highlighting the potential use of alternative ICI therapy in acquired ICI resistance. 225 In primary liver cancer, combination of anti-TIM3 with anti-PD1 is currently under investigation (NCT03680508).

## Outlook—Decision-Making Based on the TME and Challenges to Overcome

Looking back on the sheer amount of options for combination therapy, we want to emphasize on the importance of hypothesis-driven and evidence-based decisions to choose adequate therapies for individual primary liver cancer patients, which is in line with what other authors proposed. 246 291 Given the fact that advanced combinations of three substances or more are currently under investigation, the potential increase in treatment-related toxicity, immune-adverse events, and therapy costs make it even more important to identify groups of patients who are in need of complex combinations to achieve successful antitumor immunity. We and others 52 246 propose the composition of the TME to be one of the most important indicators for rational therapy decisions. While patients with a cold, immune-deserted TME might benefit from ICD induction, patients with an immunosuppressive TME might benefit from MDSC blockade. Until we can use this kind of decision-making in clinical practice, the most pressing matter to explore is not only possible therapies but especially biomarkers to validate therapy decisions. For personalized TME-based therapy decisions, in-depth analysis of the individual tumor with DNA mutation variances, RNA and protein expression profiles as well as spatial distribution of tumor cells, CAFs, and immune cells would be necessary—not only once, but repeatedly to keep track of changes occurring in response to therapy. 246 Understandably, it is currently not possible to apply these techniques to every liver cancer patient treated with ICI. Therefore, one of the most important challenges for the future of ICI combination therapy is the validation of predictive TME-based biomarkers. 292 293 Fortunately, recently published studies already started to address these demands. 206 294

In summary, ICI combination therapies are able to overcome low response rates and survival benefits of ICI monotherapy and revolutionized the treatment options for patients with advanced primary liver cancer. Personalized treatment decisions based on TME-related biomarkers might further improve prediction of therapy efficacy and thereby increase therapy responses and reduce unnecessary treatments (and their side effects).

## Notes

Lay summary Primary liver cancer is one of the most common and deadliest tumors worldwide. Most patients are diagnosed in advanced stages, which limits available treatment options. Immunotherapies that can activate the immune system to eradicate cancer cells have had tremendous success in the past decade, but response rates in liver cancer are still low. Here, we summarize the mechanisms that render liver tumors resistant to immunotherapies and how these obstacles can be overcome by combining different therapy regimens, ultimately leading to higher response rates and long-lasting antitumor immunity.
